# Mass Spectrometric ITEM-ONE and ITEM-TWO Analyses Confirm and Refine an Assembled Epitope of an Anti-Pertuzumab Affimer

**DOI:** 10.3390/biom14010024

**Published:** 2023-12-24

**Authors:** Claudia Röwer, Oladapo O. Olaleye, Rainer Bischoff, Michael O. Glocker

**Affiliations:** 1 Proteome Center Rostock, Medical Faculty and Natural Science Faculty, University of Rostock, 18057 Rostock, Germany; 2 Department of Analytical Biochemistry, Faculty of Science & Engineering, University of Groningen, 9713 AV Groningen, The Netherlands

**Keywords:** affimer, therapeutic antibody, therapeutic drug monitoring, Pertuzumab, intact transition epitope mapping, ITEM-ONE, ITEM-TWO, mass spectrometry, affinity, binding strength analysis

## Abstract

Intact Transition Epitope Mapping—One-step Non-covalent force Exploitation (ITEM-ONE) analysis reveals an assembled epitope on the surface of Pertuzumab, which is recognized by the anti-Pertuzumab affimer 00557_709097. It encompasses amino acid residues NSGGSIYNQRFKGR, which are part of CDR2, as well as residues FTLSVDR, which are located on the variable region of Pertuzumab’s heavy chain and together form a surface area of 1381.46 Å^2^. Despite not being part of Pertuzumab’s CDR2, the partial sequence FTLSVDR marks a unique proteotypic Pertuzumab peptide. Binding between intact Pertuzumab and the anti-Pertuzumab affimer was further investigated using the Intact Transition Epitope Mapping—Thermodynamic Weak-force Order (ITEM-TWO) approach. Quantitative analysis of the complex dissociation reaction in the gas phase afforded a quasi-equilibrium constant ($K_{D\;m0g}^{\#}$) of 3.07 × 10^−12^. The experimentally determined apparent enthalpy (${\Delta H}_{m0g}^{\#}$) and apparent free energy (${\Delta G}_{m0g}^{\#}$) of the complex dissociation reaction indicate that the opposite reaction—complex formation—is spontaneous at room temperature. Due to strong binding to Pertuzumab and because of recognizing Pertuzumab’s unique partial amino acid sequences, the anti-Pertuzumab affimer 00557_709097 is considered excellently suitable for implementation in Pertuzumab quantitation assays as well as for the accurate therapeutic drug monitoring of Pertuzumab in biological fluids.

## 1. Introduction

With the advent of precision medicine concepts, such as “P4 medicine” [1], both companion diagnostics (CDx; [2]) and therapeutic drug monitoring (TDM; [3]) have become of utmost importance for optimizing therapy success. With the beginning of the third millennium, immunotherapy has been added to the oncologic therapeutic arsenal to fight cancer [4] with remarkable success. Upon pre-selecting eligible patients based on molecular markers, the administration of engineered monoclonal antibodies, such as Trastuzumab [5] or Pertuzumab [6], resulted in a significant break-through in breast cancer treatment [7]. As with any medication, pharmacokinetic and pharmacodynamic properties have to be determined for therapeutic antibodies and, in conjunction with personalized medicine concepts, antibody concentrations ought to be monitored during treatment at the individual patient level. Collecting material for therapeutic drug monitoring [8] is mostly performed by ascertaining bio-fluids, such as blood, plasma or serum; hence, levels of drugs, i.e., therapeutic antibodies, in the individual’s circulation are taken as surrogate markers to estimate both dosage and re-administration time periods [9]. The isolation of therapeutic antibodies from patient blood is typically performed using affinity capture methods [10] and the choice of capturing devices includes affimers as binder molecules [11]. The accuracy of the monitoring process depends on flawless functioning of the capturing procedure, which is determined, among other factors, by the binder’s properties, such as the specificity of analyte recognition [12]. The binding specificity determination of a binder molecule requests to also ascertain the binder’s epitope on the analyte’s surface [13].

Epitope mapping, mass spectrometric methods have proven highly efficient [14]. Several “flavors” of mass spectrometric epitope mapping methods have been developed, including cross-linking chemistry [15], hydrogen-deuterium exchange (HDX) procedures [16], and epitope excision/extraction methods [14,17,18]. The precision of modern mass spectrometric epitope mapping methods reaches down to the amino acid residue level [19], i.e., to the epitope peptide’s constituents, with requested sample consumptions (microliters of picomolar solutions) remaining a negligible factor [20]. The mass spectrometry-based ITEM (Intact Transition Epitope Mapping) method, developed by the authors, facilitates epitope extraction/excision by minimizing liquid sample handling to mixing an antigen-containing or epitope peptide-containing solution with an antibody-containing solution [21,22,23] prior to or after having performed an enzymatic digestion of the antigen. The specific binding of an antigen’s epitope peptide(s) to an antibody in orthodox fashion [24,25] is unequivocally determined by mass spectrometric analyses of the mass and/or the collision-induced fragment ions of the complex-released epitope peptides’ ions. In parallel, upon stepwise increasing of the collision energy, the gas-phase binding strengths of the non-covalent immune complexes’ ions are determined [26,27], providing information about the immune complex stability.

In this project, we tested whether or not our ITEM procedures could be applied to affimer binders and, if so, whether it was possible to identify the affimer 00557_709097’s epitope on a therapeutic antibody. In addition to mapping the affimer’s epitope by ITEM-ONE [23,28], we made use of Western blot analysis results and of information on the Pertuzumab epitope region, which was available through HDX MS data [29]. The results from conventional immuno-analytical and modern mass spectrometric epitope mapping methods were complementing each other and, taken together, unfolded the full picture of an assembled (conformational) affimer epitope that encompasses the CDR2 region and its C-terminally adjacent partial constant sequence on the variable region of Pertuzumab’s heavy chain. Moreover, the gas-phase binding strength of the immune complex consisting of the anti-Pertuzumab affimer and Pertuzumab was determined using our ITEM-TWO method [22,30].

## 2. Materials and Methods

### 2.1. Preparation of Affimer, Antibody, and Peptide Solutions for Mass Spectrometry

The Pertuzumab-binding affimer 00557_709097 and Pertuzumab were obtained as solutions, dissolved in PBS, and were rebuffered into 200 mM ammonium acetate, pH 6.7, as described previously [24]. In brief, aliquots with around 60 µg of the affimer and approximately 60 µg of Pertuzumab were placed in separate centrifugal filtration devices with 10 kDa exclusion pore sizes (Merck Millipore, Carrigtwohill, Ireland). The filtration devices were filled up with 200 mM ammonium acetate, pH 6.7, to a total volume of 500 µL, each. Centrifugations were performed at 13,000 rpm for 7 min in a table centrifuge (MiniSpin, Eppendorf, Hamburg, Germany) at room temperature. The flow-through fractions were discarded and the filter devices were filled up with 450 µL 200 mM ammonium acetate, pH 6.7, each. Centrifugation/discarding/re-filling steps were repeated eight times. Afterwards, the filtration devices were placed upside down on top of new tubes and were centrifuged for 5 min at 4500 rpm. Approximately 50 µL of affimer solution or antibody solution were separately collected. The protein concentrations were determined using the Qubit^TM^ 2.0 Fluorometer assay (Invitrogen by Life technologies/Thermo Fisher Scientific, Waltham, MA, USA). 

The peptide mixture from a tryptic Pertuzumab digestion [11] was desalted and concentrated using ZipTip^®^ pipette tips (Millipore Corporation, Billerica, MA, USA) after tip conditioning. A volume of 1 µL of the Pertuzumab peptide mixture from the tryptic digest was mixed with 9 µL of 0.1% TFA. For conditioning, the ZipTips^®^ were washed twice with 10 µL 50% ACN each, and then twice with 10 µL 0.1% TFA each. For loading the peptides onto a tip, the peptide mixture was aspirated up and down ten times while keeping the tip in the solution with the diluted peptide mixture. Afterwards, the tip was washed twice with 10 µL 0.1% TFA, each. Peptides were eluted by pipetting up and down 3 µL of 80% ACN/0.1% formic acid ten times. The elution procedure was performed three times with 3 µL of 80% ACN/0.1% formic acid and the eluates were pooled. A pooled eluate was allowed to dry at room temperature and peptides were redissolved in 9 µL 200 mM ammonium acetate, pH 6.7.

Synthesized peptides of Pertuzumab partial sequences and the Histag peptide were purchased as lyophilized powders (peptides&elephants, Hennigsdorf, Germany) and dissolved in 200 mM ammonium acetate, pH 6.7, to obtain peptide concentrations of ca. 1.0 µg/µL each. The actual peptide concentrations of individual peptide solutions were determined using the Qubit^TM^ 2.0 Fluorometer assay.

### 2.2. Preparation of Immune-Complex-Containing Mixtures

To obtain a complex-containing mixture of affimer 00557_709097 and Pertuzumab, 4 µL of affimer 00557_709097 solution (0.23 µg/µL in 200 mM ammonium acetate, pH 6.7) were mixed with 2 µL of a Pertuzumab solution (0.43 µg/µL in 200 mM ammonium acetate, pH 6.7). To obtain a complex-containing mixture of affimer 00557_709097 and peptides from tryptically digested Pertuzumab, 5 µL of affimer 00557_709097 solution (0.28 µg/µL in 200 mM ammonium acetate, pH 6.7) were mixed with 2 µL of a Pertuzumab peptide mixture which had been eluted from the ZipTips^®^. A complex-containing mixture of affimer 00557_709097 and one of the synthetic peptides was prepared by mixing 10 µL of an affimer 00557_709097 solution (0.2 µg/µL in 200 mM ammonium acetate, pH 6.7) with the appropriate volumes of each of the peptide solutions (solvents were 200 mM ammonium acetate, pH 6.7) to obtain molar ratios of 1:1 (affimer:peptide). In detail: 5.01 µL of peptide aa 68–74 (0.049 µg/µL), 5.4 µL of peptide aa 54–67 (0.043 µg/µL), or 4.7 µL of peptide aa 54–74 (0.15 µg/µL) were separately added to the affimer 00557_709097 solutions. Additionally, 10 µL affimer 00557_709097 solution (0.2 µg/µL in 200 mM ammonium acetate, pH 6.7) were incubated together with 6.32 µL Histag peptide solution (0.082 µg/µL, dissolved in 200 mM ammonium acetate, pH 6.7). All mixtures were incubated at room temperature for at least 1 h prior to mass spectrometric investigations. 

### 2.3. Offline nanoESI-MS Instrument Settings and Data Acquisition 

For each mass spectrometric analysis, 3 µL of a peptide (mixture) or a protein-containing solution were loaded into a nanoESI capillary needle. Capillary needles were pulled and gold-coated in house [22]. NanoESI-MS measurements were performed on a Synapt G2-S mass spectrometer (Waters MS-Technologies, Manchester, United Kingdom) with the following measurement settings: capillary voltage, ~1.2 kV; source temperature, 40 °C; source offset voltage, 100 V; sample cone voltage, 100 V; cone gas flow, 20 l/h; purge gas flow 25 l/h; trap gas flow, 1.0 mL/min; initial trap and transfer collision cell voltage, 2 V. Measurements were acquired in positive-ion mode applying a mass window of *m*/*z* 200–4000 or 200–8000. The quadrupole mass filter was set to full transmission. The mass axis was calibrated using 1 mg/mL sodium iodide dissolved in isopropanol/water (50:50, *v*/*v*). 

For ITEM measurements of complex dissociation reactions in the gas phase the quadrupole analyzer was used to block transmission of ions with low *m*/*z* values using the following settings: M1 = 5000, dwell time and ramp time 25%; M2 = 5000, dwell time and ramp time 25%; M3 = 5000. Alternatively, the quadrupole analyzer was used to block peptide ion transmission for the ITEM measurements with affimer and synthetic peptides using the following settings: M1 = 1200, dwell time and ramp time 25%; M2 = 1200, dwell time and ramp time 25%; M3 = 1200. The instrument’s first collision cell (TRAP) was used to dissociate affimer 00557_709097—Pertuzumab or affimer 00557_709097—peptide complexes, respectively, by increasing the collision cell voltage difference (∆CV) in a stepwise manner from 2 V to 90 V. Two independent measurement series were performed.

For the ion mobility separation of unbound peptides from complexes consisting of affimer 00557_709097 and peptides derived from tryptic digestion of Pertuzumab, the following settings were used: trap gas flow, 2.0 mL/min, helium cell gas flow, 180 mL/min, and IMS gas flow 90 mL/min. Further instrument settings were as follows: trap wave velocity, 700 m/s; trap wave height 6 V; IMS wave height ramping linear: start height 900 V and end height 100 V; IMS wave velocity ramping linear: start velocity 900 m/s and end velocity 100 m/s. The following settings were used for ion mobility determinations of complexes consisting of affimer 00557_709097 and synthetic peptides: trap gas flow, 4.0 mL/min; helium cell gas flow, 180 mL/min; IMS gas flow, 50 mL/min; trap wave velocity, 700 m/s; trap wave height 20 V; IMS wave height ramping linear: start height 40 V and end height 35 V; IMS wave velocity ramping linear: start velocity 2000 m/s and end velocity 700 m/s. For the dissociation of complexes upon ion mobility separation, the instrument’s second collision cell (TRANSFER) was used with a collision cell voltage difference of 200 V for the mixture of affimer 00557_709097 and tryptic Pertuzumab peptides and of 40 V for dissociating the synthetic peptides from the affimer.

The mass spectrometry raw data have been deposited at the PRIDE [31] partner repository of the ProteomeXchange Consortium with the dataset identifier PXD043203.

### 2.4. Offline nanoESI-MS Data Analysis and Apparent Kinetic and Thermodynamic Value Calculations 

The MassLynx version 4.1 (Waters MS-Technologies) was used for mass spectrometric data analysis. The scans of each measurement or ∆CV setting were combined to generate average mass spectra. To obtain the ion signal intensities of the different components in the ITEM-TWO mass spectra of affimer 00557_709097 incubated with Pertuzumab (affimer 00557_709097, Pertuzumab, affimer 00557_709097—Pertuzumab complex) mass spectra were processed with the maximum entropy (MaxEnt) method (output mass ranges: 10,000–30,000 and 140,000–190,000, respectively; damage model: Uniform Gaussian) and centering (center method: centroid top 90%, area option). The MaxEnt process’ output is a bar spectrum with heights being proportional to the single components’ concentrations. The heights of single components were used to calculate the normalized intensities of educts (Pertuzumab with one bound affimer 00557_709097 and Pertuzumab with two bound affimers 00557_709097) at the respective collision cell voltage differences. Calculations of apparent kinetic and thermodynamic values followed published protocols [30]. In brief, the normalized intensities of educts (mean values and standard deviations of replicate measurements) were plotted against the ∆CV values and a Boltzmann curve was fitted to the data points using the Origin 2023 (10.0) software. Boltzmann curve parameters were used for calculating the equation of the tangent line along the steep part of the Boltzmann curve. The mathematical procedures for calculations of kinetic and thermodynamic values using the Eyring–Polanyi equation, the Arrhenius equation, the Gibbs–Helmholtz equation, and the van´t Hoff equation followed published protocols [30]. The percentage of the overall kinetic energy which was converted to the internal energy was set to 90%. 

### 2.5. SDS-PAGE and Western Blot Analysis 

Sodium dodecylsulfate polyacrylamide gel electrophoresis (SDS-PAGE) and Western blot analyses were performed as described [20]. In short, 12 µL (2.88 µg) intact Pertuzumab, 11 µL (5.28 µg) reduced and alkylated Pertuzumab, 6 µL (1.2 µg) recombinant human tumor necrosis factor alpha (rhTNFα), or 1 µL (3 µg) affimer 00557_709097, respectively, were filled up with water to a total volume of 20 µL, each. Then, 5 µL non-reducing sample buffer were added to the intact Pertuzumab and 5 µL reducing sample buffer were added to all other preparations prior to separation on a pre-cast SDS gel (12% Bis-Tris Gel, Invitrogen, Carlsbad, CA, USA). A volume of 3 µL of a pre-stained protein ladder (Thermo Fisher Scientific; Waltham, MA, USA) was used as an apparent molecular mass marker. After protein separation, the gel was placed in ε-aminocaproic acid buffer for 15 min until preparation of the blot. Semi dry blotting was performed onto a PVDF-FL membrane for 1 h with a constant electric current of 64 mA. After blotting, the membrane was stained with Ponceau S and then the open membrane surface was blocked with blocking buffer (Intercept blocking buffer (LI-COR Biosciences, Lincoln, NE, USA)/phosphate buffered saline 1:1) for 2 h at room temperature. The membrane was incubated with anti-Pertuzumab affimer 00557_709097 (1 µg/mL in blocking buffer with 0.1% Tween 20) for 1.5 h at room temperature, next with an anti-Histag antibody from mice (BioRad) diluted to 1:1000 with blocking buffer with 0.1% Tween 20 overnight at 4 °C. Finally, an anti-mouse antibody from goat which carried a fluorescence label (LI-COR Biosciences, Lincoln, NE, USA), diluted 1:15,000 with blocking buffer with 0.1% Tween 20, was added and incubated for one hour at room temperature. The membrane was washed 4 times for 5 min with PBS/0.1% Tween 20 after each of the antibody incubation steps. 

An inverse experiment was performed by loading an SDS gel with anti-Pertuzumab affimer 00557_709097. Protein separation and blotting onto a PVDF-FL membrane was performed as described above. Then, the membrane was cut into strips which were incubated either with 1.5 µg/mL Pertuzumab, with blocking buffer (blank), or with 1.5 µg/mL Trastuzumab (negative control) as primary antibodies which had been dissolved in blocking buffer with 0.1% Tween 20. Incubations with the primary antibodies were performed overnight at 4 °C. Afterwards, the membrane strips were washed 4 times prior to incubations with an anti-human antibody from goat which carried a fluorescence label (from Rockland Immunochemicals, Gilbertsville, PA, USA; Code: 609-132-003). Incubation time periods with the secondary antibody were 1 h. The secondary antibody was used with a dilution of 1:15,000 in blocking buffer with 0.1% Tween 20. Afterwards, membrane strips were washed 4 times. 

All membranes and strips were rinsed with PBS before scanning with the Odyssey DLx Imaging system (LI-COR Biosciences; Bad Homburg, Germany) with both excitation wavelengths of 700 nm and 800 nm in parallel. The images were converted to grey-scale images.

### 2.6. Surface Area Calculations 

The Chimera 1.14rc (UCSF) [32] software was used to visualize the Pertuzumab Fab structure model. The solvent-accessible surface area (SASA) of the epitope region was calculated using the “EpiMED-Surf” web tool (http://www.pzr.uni-rostock.de/Surfacer/ accessed on 9 December 2022) [33]. SASAs were computed for each atom (excluding H atoms) using the “rolling ball” or Shrake Rupley algorithm [34]. A probe sphere with 960 points was rolled along the van der Waals surfaces; its center depicts the SASA. The 3D coordinates of the atoms of crystallized Pertuzumab (taken from the 1S78.pdb file) were used as entry file.

## 3. Results

### 3.1. In-Solution Binding Analysis of the Anti-Pertuzumab Affimer 00557_709097 to Pertuzumab

In Western blot analyses, the SDS-PAGE-separated and blotted intact Pertuzumab, or its reduced and alkylated heavy and light chains were exposed to the anti-Pertuzumab affimer 00557_709097, which also carries a Histag. Locations of intact Pertuzumab or its heavy and light chains on the blot membrane were determined by visual inspection after Ponceau S-staining. In case the affimer’s epitope was presented after having blotted Pertuzumab or its heavy and light chains, binding of the anti-Pertuzumab affimer 00557_709097 should have been detected via the anti-Histag antibody whose presence in the complex should have led to complex formation with the fluorescence-labeled anti-mouse detector antibody from goat (Figure S1A). Decoration of the blotted Histag-carrying rhTNFα protein (positive control) indicated that the anti-Histag antibody from mouse recognized the Histag of immobilized rhTNFα and consequently the fluorescence-labeled anti-mouse detector antibody from goat was able to decorate this complex as well.

The inverse Western blot assay between the anti-Pertuzumab affimer 00557_709097 and Pertuzumab, i.e., when Pertuzumab was used as a primary antibody and the anti-Pertuzumab affimer 00557_709097 had been blotted onto the PVDF membrane after SDS-PAGE, confirmed binding. The anti-Pertuzumab affimer 00557_709097— Pertuzumab complex was visualized with a fluorescence-labeled anti-human detector antibody from goat, thereby exhibiting a strongly stained band at the location of the anti-Pertuzumab affimer 00557_709097 on the blot membrane (Figure S1B).

Both Western blot results together indicate the existence of an assembled anti-Pertuzumab affimer 00557_709097 epitope on the Pertuzumab surface, which had been distorted by SDS-PAGE and/or during blotting of Pertuzumab onto a PVDF membrane. 

### 3.2. Anti-Pertuzumab Affimer 00557_709097—Pertuzumab Complex Binding Strength Analysis by ITEM-TWO

**Figure 1:**
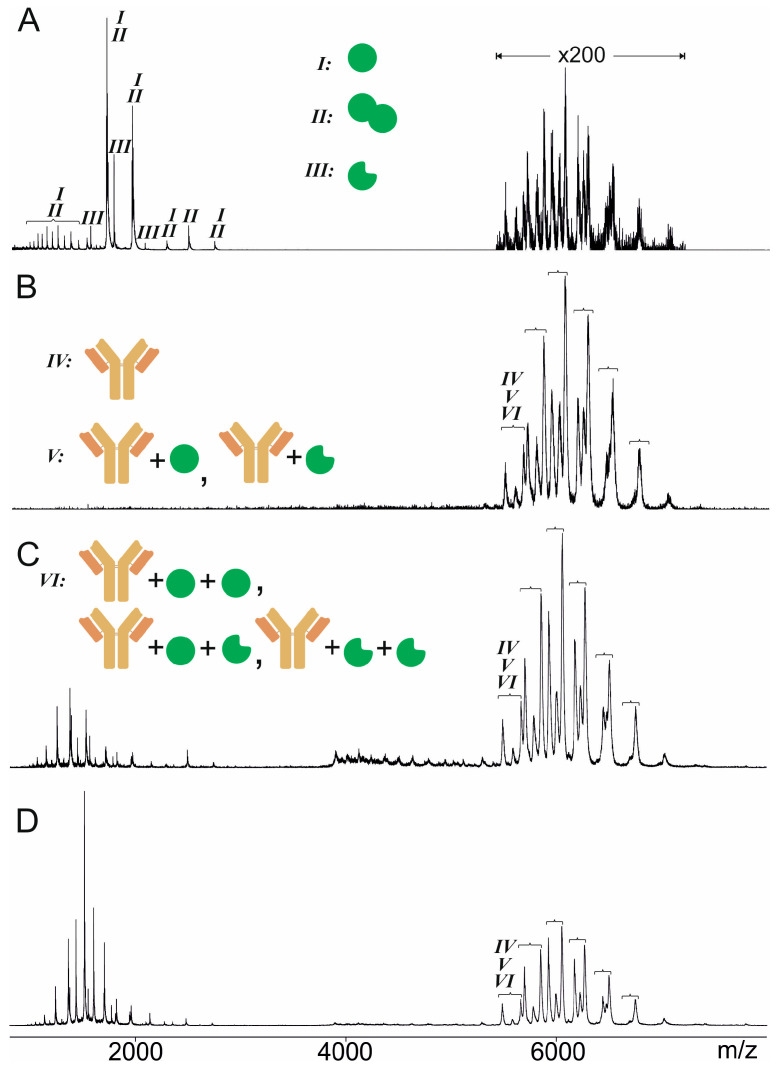
Offline nanoESI mass spectra of anti-Pertuzumab affimer 00557_709097 incubated with Pertuzumab. The molar ratio of anti-Pertuzumab affimer 00557_709097 to Pertuzumab was 12 to 1. Solvent: 200 mM ammonium acetate, pH 6.7. Ion signals are assigned to monomeric full-length affimer (green filled circle, I), dimeric full-length affimer (green filled circle tandem, II), monomeric truncated affimer (green filled circle with rectangular cutout, III), Pertuzumab (orange-brown y-shaped icon, IV), Pertuzumab + 1 monomeric full-length affimer or 1 monomeric truncated affimer (V), Pertuzumab + 2 monomeric full-length affimers, or 2 monomeric truncated affimers, or combinations thereof, or +1 dimeric full-length affimer, or 1 dimeric truncated affimer, or combinations thereof (VI). For ion signal intensities see Tables S1–S7. Roman numerals according to Table 1. (**A**) The quadrupole was set to full transmission of all ions and the Trap collision cell voltage difference was set to 2 V. (**B**) The quadrupole was set to block transmission of ions with *m*/*z* < 3850 and the Trap collision cell voltage difference was set to 2 V. (**C**) The quadrupole was set to block transmission of ions with *m*/*z* < 3850 and the Trap collision cell voltage difference was set to 40 V. (**D**) The quadrupole was set to block transmission of ions with *m*/*z* < 3850 and the Trap collision cell voltage difference was set to 60 V.

Since *in-solution* binding between the anti-Pertuzumab affimer 00557_709097 and intact Pertuzumab was proven by an immuno-analytical standard assay, we performed ITEM-TWO experiments which start with mixing two solutions, one which contains the anti-Pertuzumab affimer 00557_709097 and one which contains Pertuzumab. The recorded mass spectra revealed that the mixture of the anti-Pertuzumab affimer 00557_709097 and Pertuzumab contained more than two components (Figure 1, Table 1 and Table S1–S7), which were assigned as affimer monomer (I), affimer dimer (II), truncated affimer monomer (III), Pertuzumab (IV), Pertuzumab plus one affimer monomer (V), and Pertuzumab plus two affimer monomers (VI).

For each of the protein and complex components, the mean charge states (z) of the respective ion series were determined together with the corresponding molecular masses (MM). In addition, the atom numbers of the identified proteins and protein complexes were estimated from literature data since the amino acid sequence of the anti-Pertuzumab affimer 00557_709097 had not been disclosed (Table 1).

**Figure 2:**
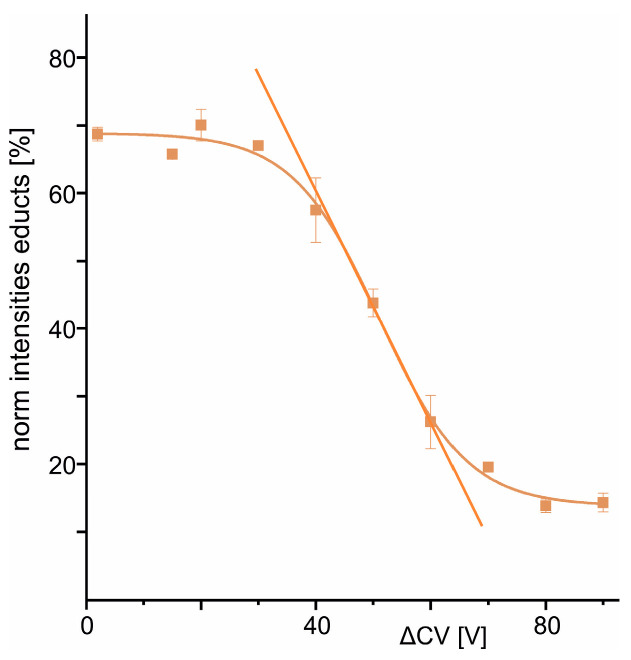
Boltzmann plot for the gas phase complex dissociation reaction of the anti-Pertuzumab affimer 00557_709097 bound to Pertuzumab. The course of normalized educt ion intensities (average of two independent measurement series) is shown as a function of collision cell voltage difference (ΔCV). Complexes consisted of (i) Pertuzumab + 1 monomeric full-length affimer or +1 monomeric truncated affimer (V) and (ii) Pertuzumab + 2 monomeric full-length affimers, or +2 monomeric truncated affimers, or combinations thereof, or of Pertuzumab + 1 dimeric full-length affimer, or +1 dimeric truncated affimer, or combinations thereof (VI). Roman numerals according to Table 1. Data points with standard deviations (vertical bars) are the means of two measurements each. The tangent line determines the linear dependency of the complex dissociation reaction in the gas phase with respect to collision cell voltage difference (ΔCV).

In the first set of ITEM-TWO experiments, the transmission of ions with low *m*/*z* (*m*/*z* < 3850) was blocked and then the collision cell voltage difference (ΔCV) in the TRAP collision cell was step-wise increased from 2 V up to 90 V to initiate dissociation of the complex’s constituents by collision induced dissociation (CID). Separate mass spectra with respect to the selected instrument’s ΔCV settings were recorded (Figure 1). Under these ion transmission blocking conditions, detected ion signals below *m*/*z* 3850 belonged to the complex-released affimer. Next, ion intensities were determined separately for educts (complexes) and products (complex-released components) of the gas phase dissociation reaction, individually for each of the recorded spectra (Supplemental Tables S8 and S9). Ion intensities from duplicate measurement series were averaged and the course of the educts’ ions’ intensities was plotted as a function of ΔCV (Figure 2).

The educt intensity diminished with increasing ΔCV and the educt ion intensity course followed a Boltzmann curve whose steep part marks the region (see tangent in Figure 2) where educt ion intensity decreases linearly with increasing collision energy. The curve’s transition point, ΔCV_50_, was reached at 51.13 V (Table 2).

The linear range of the Boltzmann curve (±dx around ΔCV_50_) marks the experimentally accessible regime of the complex dissociation reaction with the multiply charged and accelerated complex ions in the gas phase. This reaction course is converted to a temperature dependence course to calculate either the kinetic properties (Arrhenius plot, Figure S2) or the quasi thermodynamic properties (Gibbs–Helmholtz plot; Figure S3) of the monomolecular dissociation reaction. As in both cases, kinetic (k) and quasi thermodynamic (K) dependencies logarithmically follow temperature dependence, linear extrapolation of the respective logarithmic values to ambient temperature leads to the dissociation properties of a resting and neutral complex consisting of the anti-Pertuzumab affimer 00557_709097 and Pertuzumab. Using the Eyring–Polanyi equation and the van’t Hoff equation leads to the free energy (${\Delta G}_{m0g}^{\#}$), enthalpy (${\Delta H}_{m0g}^{\#}$), and entropy ($T_{amb}{\Delta S}_{m0g\;}^{\#}$) values of the gas phase dissociation reaction of the neutral and resting complex (Table 3).

As it turns out, the dissociation reaction of the neutral and resting anti-Pertuzumab affimer 00557_709097—Pertuzumab complex at room temperature is not spontaneous (positive ΔG) despite being exothermic (negative ΔH) since dissociation is accompanied by a decrease in entropy (negative TΔS). Hence, the inverse reaction, i.e., complex formation, is spontaneous (negative ΔG) at room temperature. Enthalpy costs (positive ΔH) are compensated by a larger entropy gain (positive TΔS).

### 3.3. Anti-Pertuzumab Affimer 00557_709097 Epitope Mapping by ITEM-ONE

**Figure 3:**
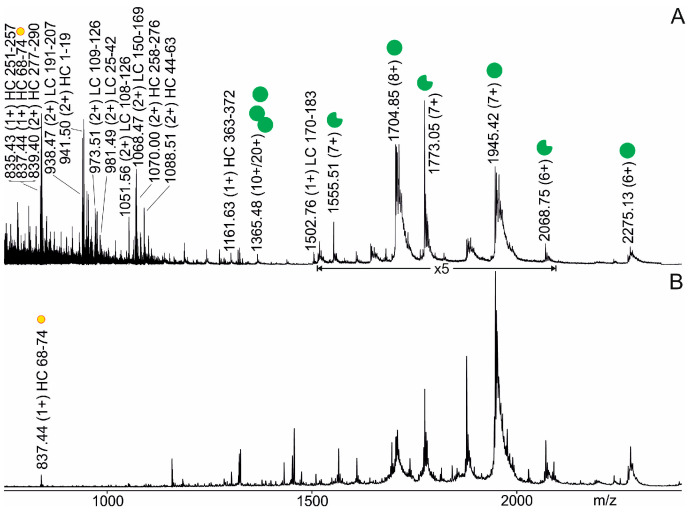
Offline nanoESI mass spectra of the tryptic peptide mixture from Pertuzumab incubated with the anti-Pertuzumab affimer 00557_709097. Selected *m*/*z* values and charge states of ion signals from the anti-Pertuzumab affimer 00557_709097 (monomeric full-length affimer (green filled circle, I), dimeric full-length affimer (green filled circle tandem, II), monomeric truncated affimer (green filled circle with rectangular cutout, III), and from peptides with amino acid ranges from Pertuzumab heavy chain (HC) or light chain (LC) are given. The complex-released epitope peptide FTLSVDR (HC 68–74) from Pertuzumab is indicated with a yellow filled circle. Solvent: 200 mM ammonium acetate, pH 6.7. Spectra were smoothed using the Savitzky Golay algorithm applying a smooth window of 10 channels and 20 numbers of smooth cycles. Roman numerals according to Table 1. (**A**) The quadrupole was set to full transmission of all ions and the Trap collision cell voltage difference was set to 2 V. (**B**) The quadrupole was set to block transmission of ions < *m*/*z* 1360 and the Trap collision cell voltage difference was set to 50 V.

**Figure 4:**
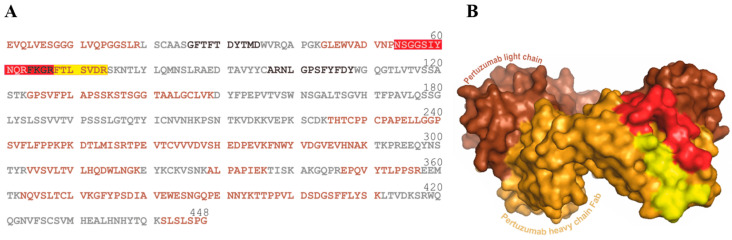
Amino acid sequence of the Pertuzumab heavy chain in single letter code and cartoon of the Pertuzumab Fab fragment showing van-der-Waals atom surfaces. (**A**) Partial peptide sequences of peptides which were obtained after tryptic Pertuzumab digestion and subsequent desalting are shown in orange (sequence coverage ~51%). Complementarity-determining regions 1, 2, and 3 [36] are printed in black letters. All other partial sequence stretches are printed in gray letters. Peptides of the anti-Pertuzumab affimer 00557_709097 binding region are marked in red (amino acid residues 54–67) and in yellow (amino acid residues 68–74), respectively. (**B**) The Pertuzumab light chain is colored in brown and the heavy chain Fab fragment in orange. The anti-Pertuzumab affimer 00557_709097 binding region is colored red (amino acid residues 54–67) and yellow (amino acid residues 68–74), respectively. The model was generated with Pymol (Version 2.5.7) using the atom coordinates from the 1S78.pdb file.

To determine which partial surface on Pertuzumab made direct contact with the anti-Pertuzumab affimer 00557_709097, an epitope extraction experiment was started by digesting Pertuzumab with trypsin. The tryptic peptide mixture needed to be filtered and rebuffered to remove the protease and to allow complex formation between the resolubilized tryptic peptides at near-neutral pH. Rebuffering caused substantial losses and only a fraction of the enzymatically produced peptides was resolubilized (Table S10). Rather poor sequence coverages of 51% for the heavy chain and 59% for the light chain were determined by nanoESI mass spectrometry (Figure 3A and Figure 4A, Table S10). Nevertheless, the complexation of tryptic Pertuzumab peptides with the anti-Pertuzumab affimer 00557_709097 afforded binding of the heavy chain partial amino acid sequence FTLSVDR (aa68–74), which was directly observed by mass spectrometric ITEM-ONE analysis (Figure 3B). The only singly protonated ion signal in the mass spectrum, which was recorded under ITEM-ONE conditions, i.e., in the mass range below *m*/*z* 1350 after the blocking of transmission of low *m*/*z* ions and upon increasing the collision cell voltage difference was the ion signal at *m*/*z* 837.44. This ion signal was hence assigned as complex-released peptide, i.e., an epitope peptide.

The ion signal at *m*/*z* 837.44 had already been recorded in the tryptic peptide mixture (Figure 3A) after rebuffering, and mass spectrometric sequencing by CID of this ion signal unambiguously determined its amino acid sequence as FTLSVDR (Figure S4).

Ion mobility drift time analysis of free tryptic peptides from Pertuzumab and of anti-Pertuzumab affimer 00557_709097, as well as complexes and by CID released peptides substantiated binding of the FTLSVDR peptide. The ion mobility drift time of the unbound FTLSVDR peptide ion was 12.57 ms whereas the drift time of the same peptide was 15.66 ms when it had bound and, thus, had been carried through the ion mobility chamber as cargo of the much larger anti-Pertuzumab affimer 00557_709097 molecule (Figure S5). The ion mobility drift time of the anti-Pertuzumab affimer 00557_709097 alone was determined to be 15.10 ms, confirming that the FTLSVDR peptide had been captured by the anti-Pertuzumab affimer 00557_709097 in solution but was released in the gas phase in the TRANSFER cell, i.e., after ion mobility separation of ions.

The X-ray structure model (1S78.pdb; Figure 4) of Pertuzumab showed that the partial sequence FTLSVDR (aa68–74; yellow in Figure 4) forms part of the heavy chain´s surface and is located next to the CDR2 loop (aa48–67; red in Figure 4) of Pertuzumab. The amino acid residues from both (aa48–74) form a surface area of 1381.46 Å^2^, which is large enough to form an assembled epitope of the anti-Pertuzumab affimer 00557_709097.

The C-terminal part of the CDR2 loop (aa54–67; red in Figure 4) had been determined as an epitope of the anti-Pertuzumab affimer 00557_709097 by H/D exchange combined with mass spectrometry as read-out. Of note, in this H/D exchange analysis, no information about shielding by complexation was obtained for the FTLSVDR partial amino acid sequence [29].

### 3.4. Anti-Pertuzumab Affimer 00557_709097 Epitope Validation with Synthetic Peptides

Since complementing epitope information for the anti-Pertuzumab affimer 00557_709097 came from two different types of analyses we conducted validation experiments with synthetic peptides. Pertuzumab partial sequences were selected, which encompassed the respective amino acid sequence stretch NSGGSIYNQRFKGR (aa54–67) which had been determined as anti-Pertuzumab affimer 00557_709097 binding region by H/D exchange, FTLSVDR (aa68–74), which had been determined as a binding region by ITEM-ONE, and NSGGSIYNQRFKGRFTLSVDR (aa54–74), which is a combination of the two aforementioned peptides (Table S11). 

**Figure 5:**
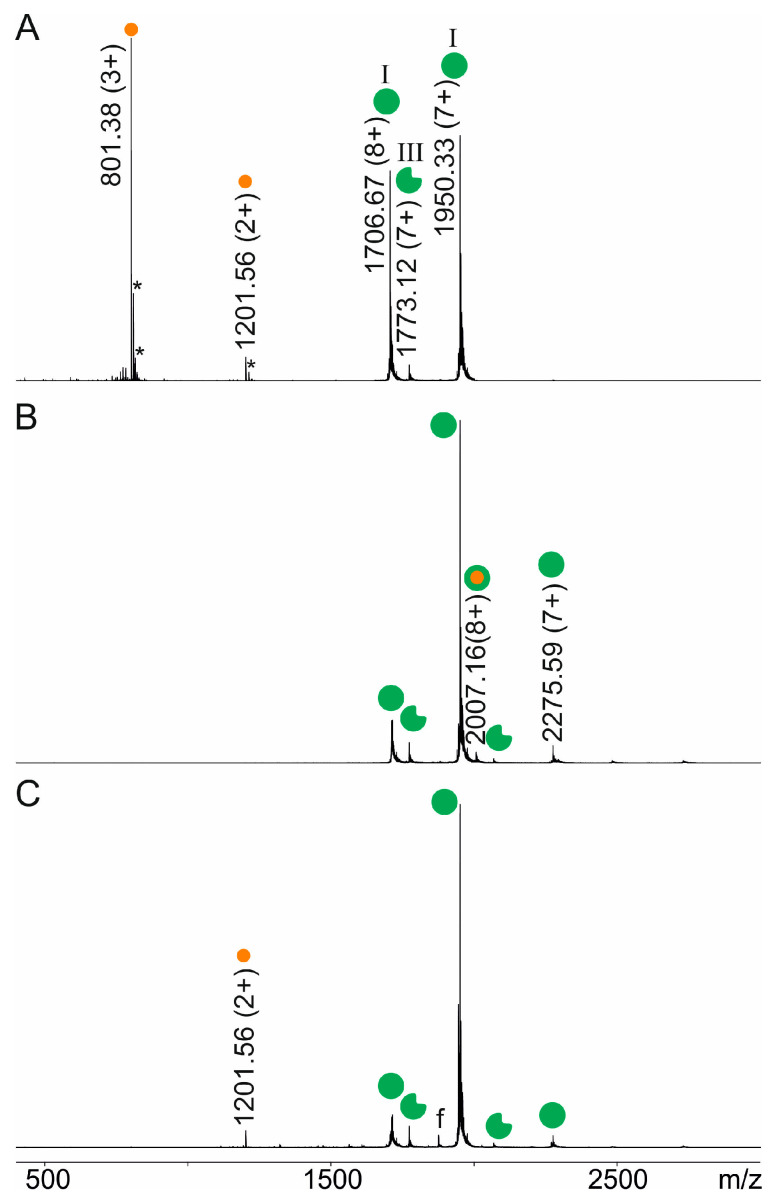
Offline nanoESI mass spectra of the NSGGSIYNQRFKGRFTLSVDR peptide from the Pertuzumab heavy chain incubated with the anti-Pertuzumab affimer 00557_709097. The molar ratio of anti-Pertuzumab affimer 00557_709097 to the NSGGSIYNQRFKGRFTLSVDR peptide was 1 to 2. Selected *m*/*z* values and charge states of ion signals are given for the anti-Pertuzumab affimer 00557_709097 (monomeric full-length affimer (green filled circle, I) and monomeric truncated affimer (green filled circle with rectangular cutout, III), and for peptide NSGGSIYNQRFKGRFTLSVDR (amino acid range 54–74 from Pertuzumab heavy chain (HC); orange filled circle). Complex ion signals are indicated with orange filled circles centered within green filled circles. f: known peptide or affimer fragment ion signals. *: sodium and/or potassium adducts. Solvent: 200 mM ammonium acetate, pH 6.7. Spectra were smoothed using the Savitzky–Golay algorithm applying a smooth window of 10 channels and 20 numbers of smooth cycles. Roman numerals according to Table 1. (**A**) The quadrupole was set to full transmission of all ions and the Trap collision cell voltage difference was set to 2 V. (**B**) The quadrupole was set to block transmission of ions < *m*/*z* 1650 and the Trap collision cell voltage difference was set to 2 V. (**C**) The quadrupole was set to block transmission of ions < *m*/*z* 1650 and the Trap collision cell voltage difference was set to 40 V.

Peptides were mixed with the anti-Pertuzumab affimer 00557_709097 one after the other with slight excesses of the peptide. ITEM-ONE analysis revealed complex formation by detecting the complex-released peptide in the low *m*/*z* region of the recorded mass spectrum. Recording the doubly protonated ion signal at *m*/*z* 1201.56 upon increasing the collision cell voltage difference determined peptide NSGGSIYNQRFKGRFTLSVDR (aa54–74) as binding to the anti-Pertuzumab affimer 00557_709097. This ion signal was the only one which was observed in the low mass range of the mass spectrum upon blocking transmission of low *m*/*z* ions and increasing the collision cell voltage difference (Figure 5).

Similarly, the binding of both shorter peptides, NSGGSIYNQRFKGR (aa54–67) and FTLSVDR (aa68–74), had been clearly confirmed by ITEM-ONE analysis (Figures S6 and S7). Again, the only singly or doubly protonated ion signals in the low *m*/*z* ranges were those of the complex-released peptides. In addition, multiply charged ion signals which belonged to the respective complexes consisting of the anti-Pertuzumab affimer 00557_709097 with one bound peptide had been observed in the high mass regions (above *m*/*z* 1650) of the recorded mass spectra independent of the quadrupole filter settings but at low collision cell voltage differences in all three cases. By contrast, no binding to the anti-Pertuzumab affimer 00557_709097 was detected when an unrelated peptide had been added. The Histag carrying peptide GSSHHHHHHSSGLVPR was chosen to serve as a negative control (Figure S8).

To complete our ITEM-ONE investigations of peptide binding to the anti-Pertuzumab affimer 00557_709097, we analyzed the ion mobility drift times of the free peptides and compared them to the drift times of the same peptides after having added the anti-Pertuzumab affimer 00557_709097 to the respective peptide in solution.

**Figure 6:**
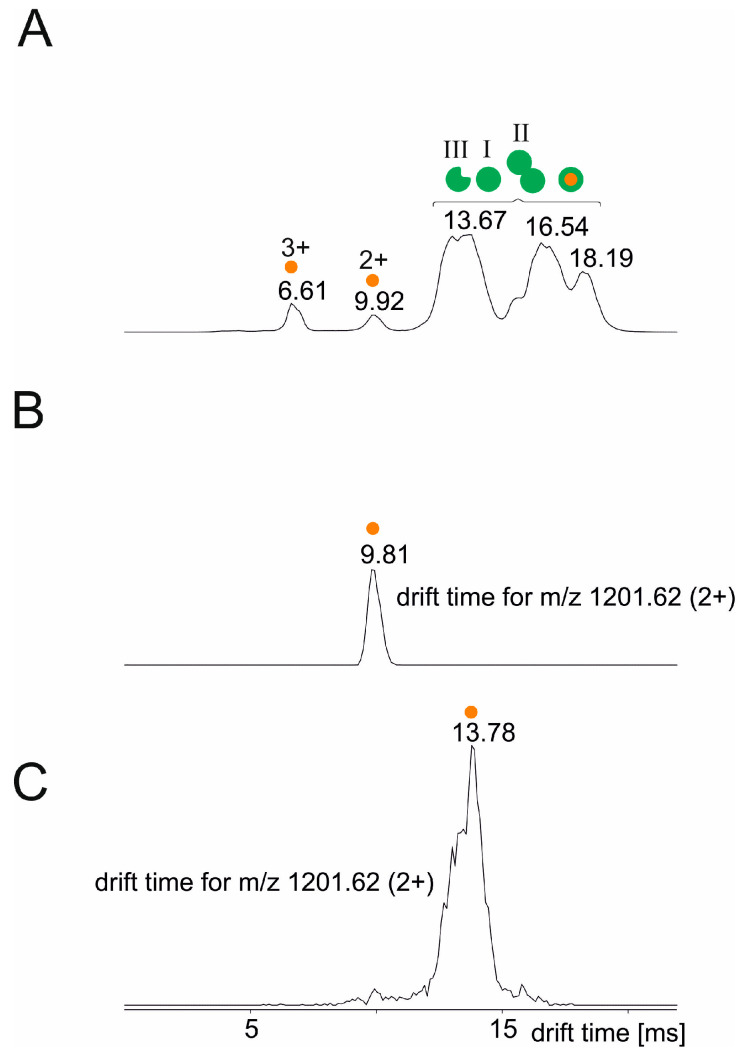
Ion mobility drift time plot of the NSGGSIYNQRFKGRFTLSVDR peptide incubated with the anti-Pertuzumab affimer 00557_709097. The molar ratio of anti-Pertuzumab affimer 00557_709097 to the NSGGSIYNQRFKGRFTLSVDR peptide was 1 to 2. Drift times are given for ions from the anti-Pertuzumab affimer 00557_709097 (monomeric full-length affimer (green filled circle, I), the dimeric full-length affimer (green filled circle tandem, II), and the monomeric truncated affimer (green filled circle with rectangular cutout, III), and for peptide NSGGSIYNQRFKGRFTLSVDR (amino acid range 54–74 from Pertuzumab heavy chain (HC); orange filled circle). Complex ions are indicated with orange filled circles centered within green filled circles. Traces were smoothed using the Savitzky–Golay algorithm applying a smooth window of 2 channels and 2 smooth cycles. Roman numerals according to Table 1. (**A**) Drift time plot for all ions at Transfer collision cell voltage difference of 2V. (**B**) Drift time plot for ion with *m*/*z* value of 1201.62 (doubly protonated NSGGSIYNQRFKGRFTLSVDR peptide) at Transfer collision cell voltage difference of 2 V. (**C**) Drift time plot for ion with *m*/*z* value 1201.62 at Transfer collision cell voltage difference of 75 V.

As expected, the ion mobility drift time of free peptide NSGGSIYNQRFKGRFTLSVDR (aa54–74) depended on its protonation state. The triply protonated ion´s drift time was 6.61 ms and that of the doubly protonated ion was 9.92 ms (Figure 6 and Table 4). The drift time of the anti-Pertuzumab affimer 00557_709097 was again above 13 ms. For the anti-Pertuzumab affimer 00557_709097 and its complex, we observed three drift time maxima which were partially resolved, indicating higher oligomeric affimer structures and/or drift time differences of different charge states. Most interestingly, the ion mobility drift time of peptide NSGGSIYNQRFKGRFTLSVDR (aa54–74), which was captured by the anti-Pertuzumab affimer 00557_709097 in solution and released from the complex in the gas phase by increasing the collision cell voltage difference in the TRANSFER cell, was 13.78 ms (Table 4). This drift time value matched that of the anti-Pertuzumab affimer 00557_709097. 

Similar ion mobility drift time courses were recorded when either peptide NSGGSIYNQRFKGR (aa54–67) or peptide FTLSVDR (aa68–74) had been added to the anti-Pertuzumab affimer 00557_709097 in solution (Figures S9 and S10 and Table 4). Thus, the capture of these shorter peptides by the anti-Pertuzumab affimer 00557_709097 was confirmed. 

Finally, the Histag-carrying peptide GSSHHHHHHSSGLVPR was again applied as a negative control and, as expected, showed no shift in its ion mobility drift time, no matter whether or not the peptide was electrosprayed from a solution which contained the anti-Pertuzumab affimer 00557_709097 (Figure S11 and Table 4). Instead, under the chosen experimental conditions, the Histag carrying peptide showed some fragmentation under higher collision cell voltage differences, which is typically seen for fragile and unbound peptides, whereas complex-released peptides—fragile or not—generally do not fragment well in the collision cell because of adiabatic cooling upon release from the complex.

## 4. Discussion

Contrary to many other mass spectrometry-coupled epitope mapping procedures, such as HDX MS [16,37], FPOP [38], or cross-linking [15,39], there is no need for chemical labeling of the antigen to determine the epitope with the here applied ITEM-ONE method [23]. Also different from hitherto mostly applied epitope extraction and epitope excision methods [40] or peptide chip-based epitope mapping approaches [41,42], with ITEM-ONE there is no need for immobilization of neither the capture molecule (e.g., an antibody) [43,44] nor the antigen [45]. Forming a stable in-solution complex between antigen and binder in an ESI-MS compatible buffer is sufficient for ITEM analyses.

Contrary to many methods which detect the presence of a protein complex, with ITEM-TWO there is no need for the use of additional antibodies which carry labels to positively confirm complex formation, as is the case, e.g., with conventional immuno-analytical methods like Western blot [46], FACS [47], and ELISA [48]. Instead, ITEM-TWO makes use of the strength of native mass spectrometry [49] in this respect.

Contrary to surface plasmon resonance or related methods [50] for determining affinities, ITEM-TWO requests no chemical immobilization of one of the binding partners, making quantitative binding analysis very simple and perhaps least error prone. In addition, performing ITEM-ONE and ITEM-TWO is fast and typically a series of measurements including blanks and negative controls is done within a few hours [51]. Also, compared to all the other above mentioned methods, ITEM-ONE and ITEM-TWO need very little material. Typically, a few micrograms (femto to pico moles) of antigen and binder macro-molecule are consumed [21].

There are two possible scenarios for mapping an epitope by analyzing an antigen’s peptides. Whether or not it is advised to add the protease before or after complex formation depends on the proteolysis resistances of the complex-forming partners. Epitope excision is successful when the binder but not the antigen is resistant to digestion and, hence, the binder retains its complex-forming capacity in the presence of the protease while the protease-susceptible antigen gets cleaved—except for surface regions of the antigen which are protected through complexation. This prerequisite is fulfilled by many antibodies [14,17,18] or by aptamers [52], but not necessarily by affimers. As shown here, the protease which was applied to digest the antigen can efficiently be removed from the peptide mixture prior to epitope extraction, thereby nullifying the threat of harming the affimer protein structure and, hence, the binder´s function. However, by implementing an additional fractionation step within a given work-flow, there is a risk of losing parts of the to-be-analyzed mixture components. So, the subsequently obtained analysis results may be incomplete. Yet, taking sequence coverage as a measure for estimating analytical success ought to be regarded with caution for epitope extraction because determining of just one peptide as belonging to the complex is enough for defining a consecutive (linear) epitope [53] and determining two peptides as belonging to a complex is sufficient for identifying an assembled (conformational) epitope [21]. As was shown with the HDX experiments, a binding region on the Pertuzumab surface which interacted with the anti-Pertuzumab affimer 00557_709097 escaped detection despite the rather high sequence coverage of 72% [29]. On the contrary, ITEM-ONE-identified epitope peptides are determined through peptide ion signal recording after complex dissociation, independent from sequence coverage, as is shown with the here-presented results as well. Because no method is without bias and, in that respect, mass spectrometry is not different to other techniques, such as NMR- or X-ray crystallography-based structure characterization methods, a combination of methods is advised to come to comprehensive results.

The lack of obtaining atom resolution by ITEM-ONE or ITEM-TWO, the hallmark of NMR [54] and X-ray [55] when applied as epitope mapping methods in those cases where sample consumption is not an issue, may be compensated when determining interacting partners’ surfaces (i) by accompanied molecular dynamics simulations [24], (ii) by in-silico docking experiments with complex partners [56], or (iii) by adding structure prediction algorithms, such as Alphafold [57] and PepFold [58]. 

The finding that dissociation of the affimer–Pertuzumab complex in the gas phase yields in loss of entropy merits some attention. We assume that the affimer–ligand complex with its relatively large surface—compared to that of the free ligand—provides ample space for protons which had been taken up during the ESI ionization process in the ion source. Protons can move around freely and this causes that protons may be somewhat depleted on the ligand at times [59]. Then we hypothesize that this allows the bound ligand, though being part of the complex, to also vibrate in a less restrained fashion during those times when charge repulsion is absent or diminished. Hence, despite being bound, the bound ligand may adopt somewhat more collapsed conformations (or regions of somewhat more collapsed conformations), which may alter with somewhat more extended conformations (or regions of somewhat more extended conformations) more often over time, as compared to the movements of the free and protonated ligand. A complex-released ligand (at least that fraction which is recorded by mass spectrometry) has taken some of those protons from the complex upon dissociation. Since the ligand´s surface is smaller than that of the complex there supposedly are somewhat closer restrictions on the protons’ movements. Proton locations now may keep the ligand for longer times and/or more often in a more extended state to sufficiently separate the charges. As a result, the released ligand experiences reduced flexibility as compared to the complex-bound ligand which, thus, should be interpreted as a loss of entropy upon dissociation.

Of note, the roles of specific interacting chemical groups of the amino acid side chains, i.e., the molecular recognition code, can be investigated by ITEM-FOUR with applying synthetic peptides with precisely defined amino acid substitutions [19]. Likewise, with chemical modifications, e.g., phosphorylation of amino acid side chains within the epitope region [25], information on modified functional groups of distinct amino acid residues may be collected. With both approaches, passively participating surface exposed amino acid residues can be differentiated from actively in binding involved amino acid residues within the interacting molecular surfaces from two molecules [27].

In-solution determination of thermodynamic properties of complex formation may be obtained with isothermal titration calorimetry analyses [19], which typically consume several milliliters per measurement series, and which therefore was beyond the scope of this study. The very strong in-solution binding of affimers to Pertuzmab had been previously reported [11] and stands in agreement with the results from our work.

Precision medicine links patients’ needs, health care providers’ abilities, clinical laboratories’ performances, and researchers’ goals for developing custom-tailored therapies to the very patient upon accurate diagnostics of individual molecular parameters [60]. To achieve this goal of determining the genetic make-up of the individual is just the starting point. To make precision medicine work, one needs to go beyond genomics and is advised to place precision analytics in the center of interest to obtain time-resolved molecular information of the respective physiological or pathological situation. Immuno-analytical techniques hold the promise to perform the requested challenging tasks [61], and new types of specific binders, such as affimers, shall unquestionably find their position in the toolbox, which should be well equipped for finding the indicators of specific illnesses or the markers that inform the physician whether or not a drug has reached its optimal treatment levels, i.e., the optimal concentrations in the patient’s blood, in other words: the targeted therapeutic signature [62]. 

While, as a basic analytical methodology, ITEM may be seen as of help for therapeutic drug monitoring (TDM) and outperforming the more currently applied methods, it remains to be shown to what extent it may be actually implemented in a clinical setting.

## Figures and Tables

**Table 1:** Molecular information of complexes and complex constituents.

Complex/Protein ^(a)^	MM (exp.)	Mean z	Atom no. ^(b)^
Pertuzumab + 2 affimer monomers (VI)	175,442.28 ± 21.53	28.64 +	23,600
Pertuzumab + 1 affimer monomer (V)	161,817.82 ± 41.42	26.60 +	21,800
Pertuzumab (IV)	148,111.39 ± 10.62	24.92 +	20,000
affimer monomer (I)	13,645.66 ± 0.72	9.18 +	1800
truncated affimer monomer (III) ^(c)^	12,405.00 ± 0.53	8.19 +	n.d. ^(d)^
affimer dimer (II)	27,293.35 ± 3.89	17.79 +	3600
truncated affimer dimer (VII)	23,522.94 ± 0.95	11.99 +	n.d. ^(c)^

^(a)^ Roman numerals according to Figure 1 and to Tables S1–S7. ^(b)^ Rounded values. Number of atoms for Pertuzumab is set to 20,000 [19]. Number of atoms for one affimer monomer is set to 1800 [35]. ^(c)^ truncations are results of in-solution hydrolysis prior to mass spectrometric analyses. ^(d)^ n.d.: not determined.

**Table 2:** Course characteristics of gas phase dissociations of the complexes consisting of anti-Pertuzumab affimer 00557_709097 monomers and Pertuzumab.

Initial [%] ^(a)^	Final [%] ^(b)^	∆CV_50_ [V]	dx [V]	Slope [%/V]	R^2^
68.87	13.80	51.13	7.61	−1.81	0.996

^(a)^ relative complex amount at lowest applied ∆CV ^(b)^ relative complex amount at highest applied ∆CV.

**Table 3:** Apparent kinetic and quasi thermodynamic values for affimer monomer—Pertuzumab complex dissociation in the gas phase.

$k_{D\;m0g}^{\#}$ [1/s]	$K_{D\;m0g}^{\#}$ [Ø] ^(a)^	$\Delta G_{m0g}^{\#}$ [kJ/mol]	$\Delta H_{m0g}^{\#}$ [kJ/mol]	$T_{amb}\Delta S_{m0g\;}^{\#}$ ${[\mathrm{kJ}/\mathrm{mol}]\;}^{\left(b\right)}$
9.58 × 10^9^	3.07 × 10^−12^	65.21	−2.83	−67.52

^(a)^ unitless number ^(b)^ T_amb_: 298 K.

**Table 4:** Drift times of free and complexed epitope peptides and of the anti-Pertuzumab affimer 00557_709097.

Peptide Sequence	Charge State	Drift Time [ms]	
		Free Peptide	Complexed Peptide
NSGGSIYNQRFKGR	2+	7.39	14.33
FTLSVDR	1+	10.36	15.77
NSGGSIYNQRFKGRFTLSVDR	2+	9.81	13.76
GSSHHHHHHSSGLVPR	2+	7.61	n.a. ^(a)^

^(a)^ not applicable.

## Data Availability

The mass spectrometry raw data have been deposited at the PRIDE partner repository of the ProteomeXchange Consortium with the dataset identifier PXD043203.

## Supplementary Materials

The following supporting information can be downloaded at: https://www.mdpi.com/article/10.3390/biom14010024/s1, Figure S1: Western blot analyses of the anti-Pertuzumab affimer 00557_709097 binding to Pertuzumab; Figure S2: Arrhenius plot for the gas phase complex dissociation reaction of the anti-Pertuzumab affimer 00557_709097 bound to Pertuzumab; Figure S3: Gibbs-Helmholtz plot for the gas phase complex dissociation reaction of the anti-Pertuzumab affimer 00557_709097 bound to Pertuzumab; Figure S4: NanoESI mass spectrum of by CID fragmented peptide FTLSVDR from the tryptic Pertuzumab peptide mixture; Figure S5: Ion mobility drift time plot of the tryptic peptide mixture from Pertuzumab incubated with the anti-Pertuzumab affimer 00557_709097; Figure S6: Offline nanoESI mass spectra of the FTLSVDR peptide from the Pertuzumab heavy chain incubated with the anti-Pertuzumab affimer 00557_709097; Figure S7: Offline nanoESI mass spectra of the NSGGSIYNQRFKGR peptide from the Pertuzumab heavy chain incubated with the anti-Pertuzumab affimer 00557_709097; Figure S8: Offline nanoESI mass spectra of the GSSHHHHHHSSGLVPR His-tag peptide incubated with the anti-Pertuzumab affimer 00557_709097; Figure S9: Ion mobility drift time plot of the FTLSVDR peptide incubated with the anti-Pertuzumab affimer 00557_709097; Figure S10: Ion mobility drift time plot of the NSGGSIYNQRFKGR peptide incubated with the anti-Pertuzumab affimer 00557_709097; Figure S11: Ion mobility drift time plot of the GSSHHHHHHGLVPR His-tag peptide incubated with the anti-Pertuzumab affimer 00557_709097. Table S1: Ion charge states, *m*/*z* values, intensities, and experimentally determined molecular masses of the complex consisting of Pertuzumab with two affimer monomers; Table S2: Ion charge states, *m*/*z* values, intensities, and experimentally determined molecular masses of the complex consisting of Pertuzumab with one affimer monomer; Table S3: Ion charge states, *m*/*z* values, intensities, and experimentally determined molecular masses of Pertuzumab; Table S4: Ion charge states, *m*/*z* values, intensities, and experimentally determined molecular masses of the affimer monomer; Table S5: Ion charge states, *m*/*z* values, intensities, and experimentally determined molecular masses of the affimer dimer; Table S6: Ion charge states, *m*/*z* values, intensities, and experimentally determined molecular masses of the truncated affimer monomer; Table S7: Ion charge states, *m*/*z* values, intensities, and experimentally determined molecular masses of the truncated affimer dimer; Table S8: Protein ion intensities at measured collision cell voltage differences for measurement 1 of affimer 00557_709097 incubated with Pertuzumab; Table S9: Protein ion intensities at measured collision cell voltage differences for measurement 2 of affimer 00557_709097 incubated with Pertuzumab; Table S10: Assigned Pertuzumab peptide ions upon tryptic digestion and desalting and unassigned peptide ion signals; Table S11: Molecular information on synthetic peptides.
