# Supplementary material for: Mass Spectrometric ITEM-ONE and ITEM-TWO Analyses Confirm and Refine an Assembled Epitope of an Anti-Pertuzumab Affimer
Source: Biomolecules. 2023 Dec 24;14(1):24. doi: 10.3390/biom14010024 (PMC10813730; doi:10.3390/biom14010024)
Supplement: Supplementary file 1 [file biomolecules-14-00024-s001.zip › biomolecules-2697210-supplementary.pdf]

Mass spectrometric ITEM-ONE and ITEM-TWO Analyses Confirm and Refine an Assembled Epitope of an anti-Pertuzumab Affimer

Claudia Röwer [1], Oladapo Olaleye [2], Rainer Bischoff [2], and Michael O. Glocker [1,#]

1) Proteome Center Rostock, Medical Faculty and Natural Science Faculty, University of Rostock, Schillingallee 69, 18057 Rostock, Germany
2) Department of Analytical Biochemistry, Faculty of Science & Engineering, University of Groningen, Antonius Deusinglaan 1, 9713 AV Groningen, The Netherlands

Supplement

# to whom correspondence may be addressed
Michael O. Glocker, Proteome Center Rostock, University of Rostock,
Schillingallee 69, D-18057 Rostock, Germany.
Phone: +49-381-494 4930
Fax: +49-381-494 4932
E-Mail: michael.glocker@med.uni-rostock.de

Supplemental Figures

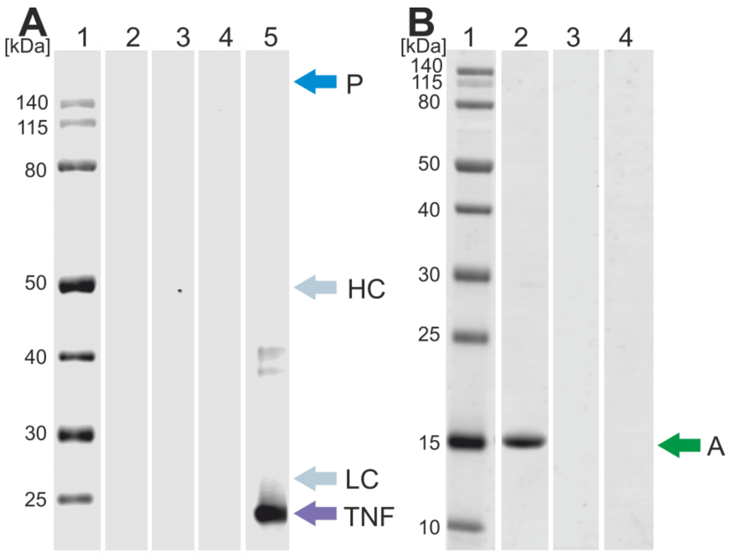

Figure S1: Western blot analyses of the anti-Pertuzumab affimer 00557_709097 binding to Pertuzumab.
A: Prestained protein ladder (PageRuler Prestained Protein Ladder; 3 µl) as apparent molecular mass marker (lane 1). Apparent molecular masses are shown at the left. 2.88 µg of intact Pertuzumab (lane 2); 5.28 µg of reduced and alkylated Pertuzumab (lanes 3 and 4) were loaded. 1.2 µg of Histag-labeled rhTNFα (lane 5) were loaded. The anti-Pertuzumab affimer 00557_709097 was used as primary antibody and serves as His-tag carrying binder. An anti-Histag antibody from mouse (BioRad) was used as secondary antibody. An anti-mouse antibody from goat labelled with IRDye® 800 was added for visualization. The location of intact Pertuzumab (lane 2; P), of heavy chains and light chains (lanes 3 and 4; HC and LC) are indicated by blueish arrows on the right. The location of the rhTNFα monomer is indicated by the purple arrow (lane 5; TNF).
B: Prestained protein ladder (PageRuler Prestained Protein Ladder; 3 µl) as apparent molecular mass marker (lane 1) and anti-Pertuzumab affimer 00557_709097 (3 µg per lane) were loaded on an SDS polyacrylamide gel (lanes 2-4). Upon addition of primary and secondary antibodies, proteins were visualized as bands. **Lane 1:** Prestained protein ladder. Apparent molecular masses are shown at the left. **Lane 2:** Pertuzumab (1.5 µg/ml) was used as primary antibody and anti-human antibody from goat labelled with IRDye® 800 was applied as secondary antibody. **Lane 3:** The primary antibody was omitted but anti-human antibody from goat labelled with IRDye® 800 was added as secondary antibody (blank). **Lane 4:** Trastuzumab (1.5 µg/ml) was used as primary antibody and anti-human antibody from goat labelled with IRDye® 800 was added as secondary antibody (negative control). The green arrow on the right points to the location of the anti-Pertuzumab affimer 00557_709097 (A) in the gel. The blot images have been cropped above 180 kDa and below 10 kDa, respectively.

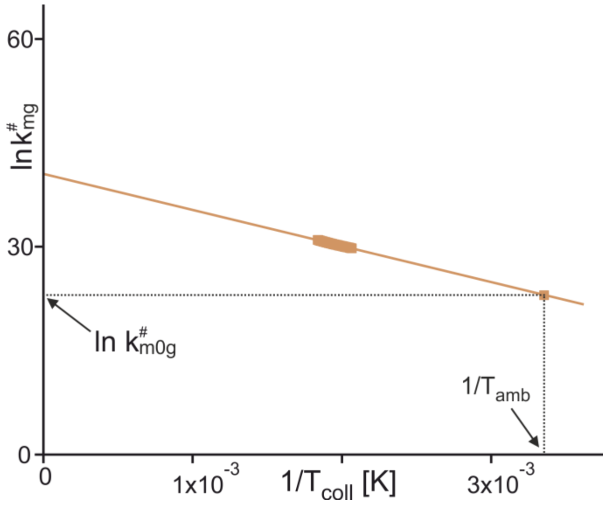

Figure S2: Arrhenius plot for the gas phase complex dissociation reaction of the anti-Pertuzumab affimer 00557_709097 bound to Pertuzumab.
Each data point (thickened part of the line) has been obtained from the linear part of the Boltzmann curve and the resulting line has been extrapolated in both directions. The value for $\boldsymbol{ln\ k^{\#}_{D\ m0g}}$ corresponds to $^{1}/_{T_{amb}}$. Calculated kinetic values are listed in Table 3.

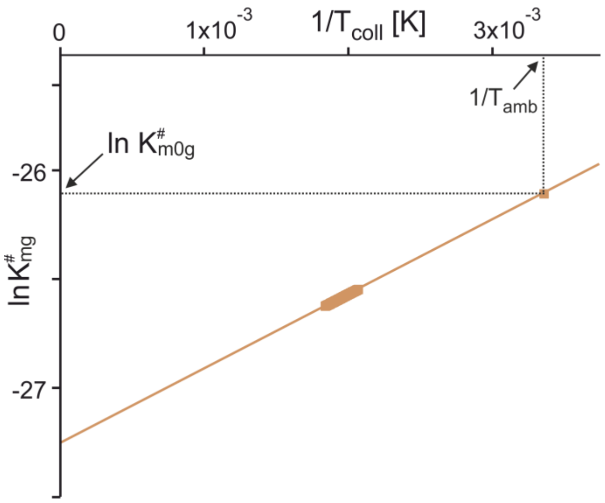

Figure S3: Gibbs-Helmholtz plot for the gas phase complex dissociation reaction of the anti-Pertuzumab affimer 00557_709097 bound to Pertuzumab.
Each data point (thickened part of the line) has been obtained from the linear part of the Boltzmann curve and the resulting line has been extrapolated in both directions. The value for $\boldsymbol{ln\ k^{\#}_{D\ m0g}}$ corresponds to $^{1}/_{T_{amb}}$. Calculated thermodynamic values are listed in Table 3.

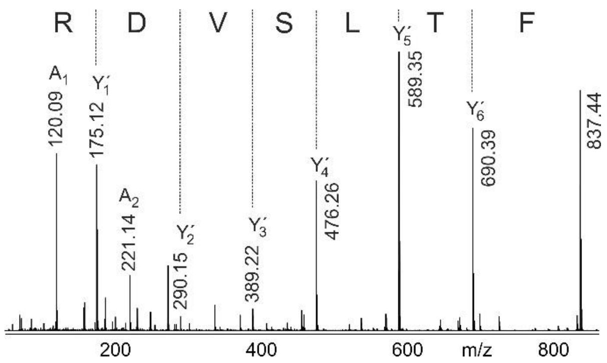

Figure S4: NanoESI mass spectrum of by CID fragmented peptide FTLSVDR from the tryptic Pertuzumab peptide mixture.
Ion signals are labelled with m/z values and fragment assignment using the nomenclature suggested by Fohlman and Roepstorff. The determined amino acid sequence of the precursor peptide with m/z 837.44 is shown in reverse order on top.

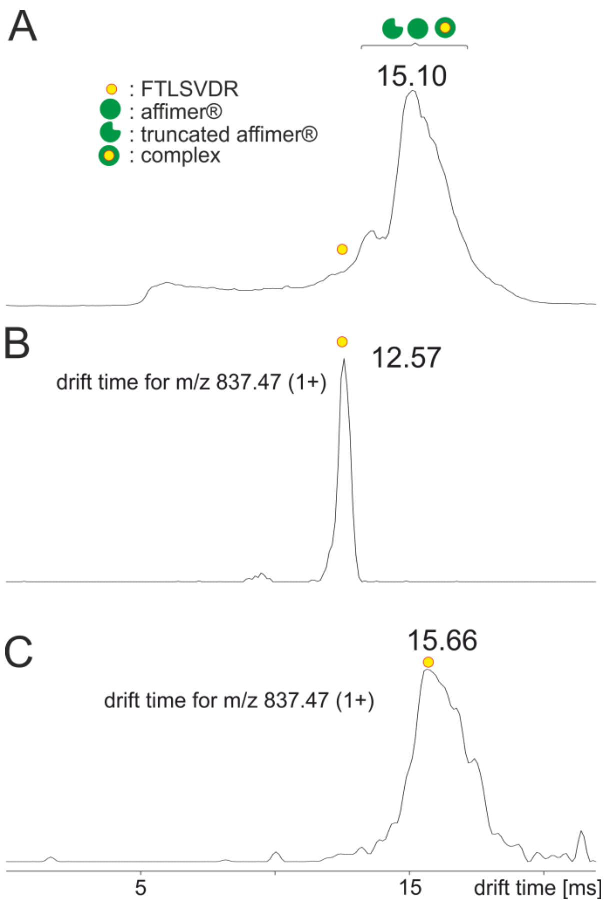

Figure S5: Ion mobility drift time plot of the tryptic peptide mixture from Pertuzumab incubated with the anti-Pertuzumab affimer 00557_709097.
Drift times are given for ions from the anti-Pertuzumab affimer 00557_709097 (monomeric full-length affimer (green filled circle, ***I***) the monomeric truncated affimer (green filled circle with rectangular cutout, ***III***), and for peptide FTLSVDR (amino acid range 68-74 from Pertuzumab heavy chain (HC); yellow filled circle). Complex ion signals are indicated with yellow filled circles centered within green filled circles. Traces were smoothed using the Savitzky Golay algorithm applying a smooth window of 2 channels and 2 smooth cycles. **A:** Drift time plot for all ions at Transfer collision cell voltage difference of 2V. **B:** Drift time plot for ion with m/z value of 837.47 (singly protonated FTLSVDR peptide) at Transfer collision cell voltage difference of 2 V. **C:** Drift time plot for ion with m/z value 837.47 at Transfer collision cell voltage difference of 200 V.

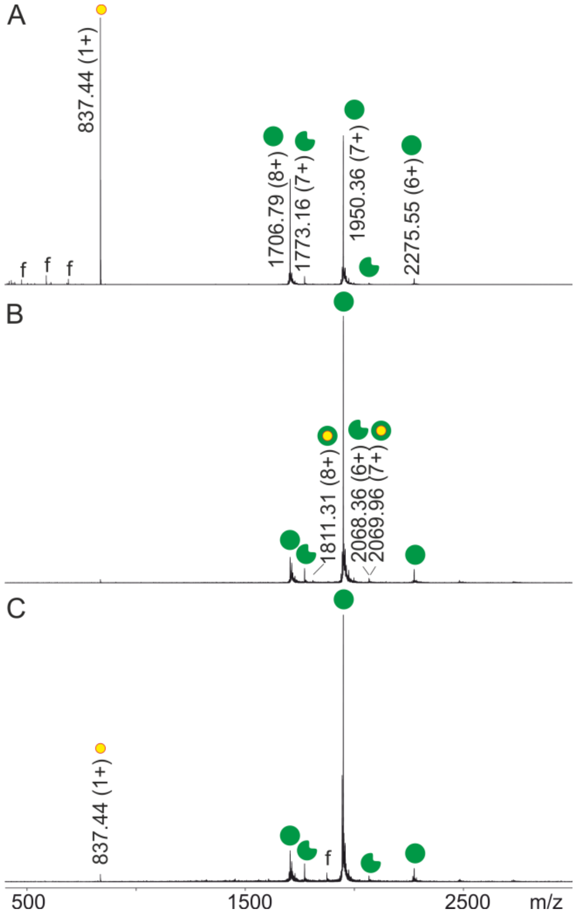

Figure S6: Offline nanoESI mass spectra of the FTLSVDR peptide from the Pertuzumab heavy chain incubated with the anti-Pertuzumab affimer 00557_709097.
The molar ratio of anti-Pertuzumab affimer 00557_709097 to the FTLSVDR peptide was 1 to 2. Selected m/z values and charge states of ion signals are given for the anti-Pertuzumab affimer 00557_709097 (monomeric full-length affimer (green filled circle, ***I***) and monomeric truncated affimer (green filled circle with rectangular cutout, ***III***), and for peptide FTLSVDR (amino acid range 68-74 from Pertuzumab heavy chain (HC); yellow filled circle). Complex ion signals are indicated with yellow filled circles centered within green filled circles. f: known peptide or affimer fragment ion signals. Solvent: 200 mM ammonium acetate, pH 6.7. Spectra were smoothed using the Savitzky Golay algorithm applying a smooth window of 10 channels and 20 numbers of smooth cycles. **A:** The quadrupole was set to full transmission of all ions and the Trap collision cell voltage difference was set to 2 V. **B:** The quadrupole was set to block transmission of ions < m/z 1650 and the Trap collision cell voltage difference was set to 2 V. **C:** The quadrupole was set to block transmission of ions < m/z 1650 and the Trap collision cell voltage difference was set to 40 V.

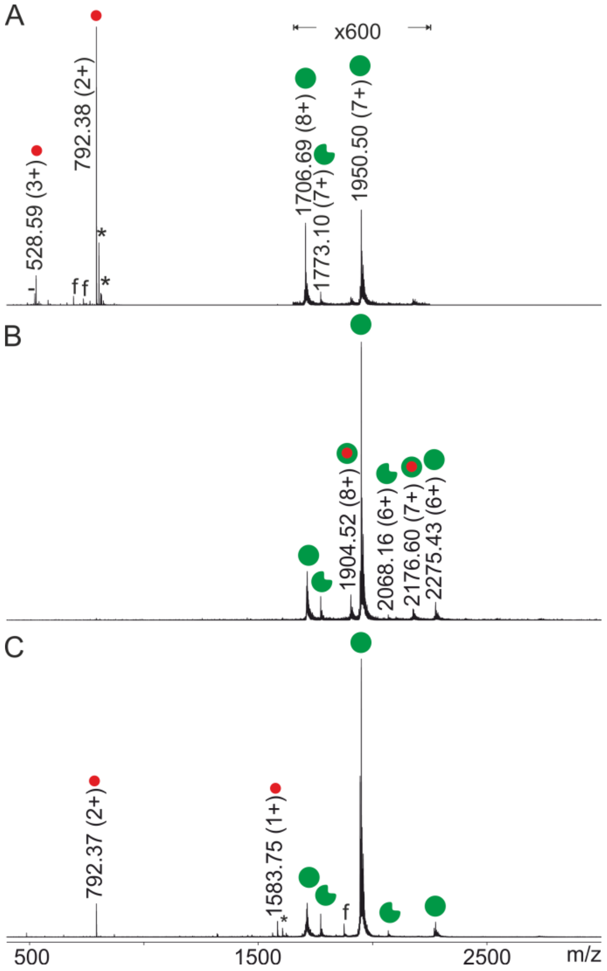

Figure S7: Offline nanoESI mass spectra of the NSGGSIYNQRFKGR peptide from the Pertuzumab heavy chain incubated with the anti-Pertuzumab affimer 00557_709097.
The molar ratio of anti-Pertuzumab affimer 00557_709097 to the FTLSVDR peptide was 1 to 2. Selected m/z values and charge states of ion signals are given for the anti-Pertuzumab affimer 00557_709097 (monomeric full-length affimer (green filled circle, I) and monomeric truncated affimer (green filled circle with rectangular cutout, III), and for peptide NSGGSIYNQRFKGR (amino acid range 54-67 from Pertuzumab heavy chain (HC); red filled circle). Complex ion signals are indicated with red filled circles centered within green filled circles. f: known peptide or affimer fragment ion signals. *: sodium and/or potassium adducts, -: loss of NH2. Solvent: 200 mM ammonium acetate, pH 6.7. Spectra were smoothed using the Savitzky Golay algorithm applying a smooth window of 10 channels and 20 numbers of smooth cycles. Ion signal multiplication factors are given. A: The quadrupole was set to full transmission of all ions and the Trap collision cell voltage difference was set to 2 V. B: The quadrupole was set to block transmission of ions < m/z 1650 and the Trap collision cell voltage difference was set to 2 V. C: The quadrupole was set to block transmission of ions < m/z 1650 and the Trap collision cell voltage difference was set to 40 V.

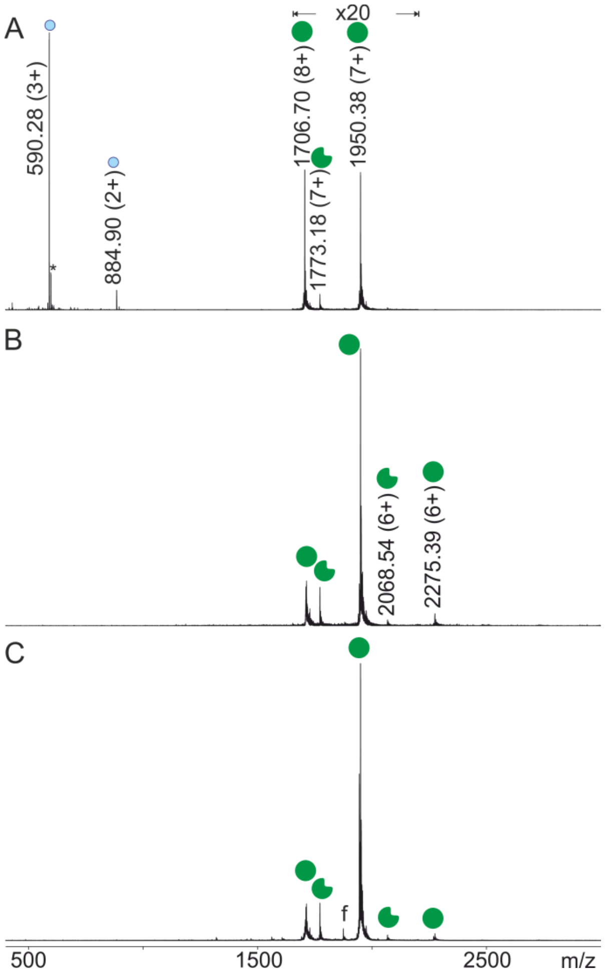

Figure S8: Offline nanoESI mass spectra of the GSSHHHHHHSSGLVPR His-tag peptide incubated with the anti-Pertuzumab affimer 00557_709097.
The molar ratio of anti-Pertuzumab affimer 00557_709097 to the GSSHHHHHHSSGLVPR peptide was 1 to 2. Selected m/z values and charge states of ion signals are given for the anti-Pertuzumab affimer 00557_709097 (monomeric full-length affimer (green filled circle, ***I***) and monomeric truncated affimer (green filled circle with rectangular cutout, ***III***), and for peptide GSSHHHHHHSSGLVPR (light blue filled circle). f: known peptide or affimer fragment ion signals. *: sodium and/or potassium adducts. Solvent: 200 mM ammonium acetate, pH 6.7. Spectra were smoothed using the Savitzky Golay algorithm applying a smooth window of 10 channels and 20 numbers of smooth cycles. **A:** The quadrupole was set to full transmission of all ions and the Trap collision cell voltage difference was set to 2 V. **B:** The quadrupole was set to block transmission of ions < m/z 1650 and the Trap collision cell voltage difference was set to 2 V. **C:** The quadrupole was set to block transmission of ions < m/z 1650 and the Trap collision cell voltage difference was set to 40 V.

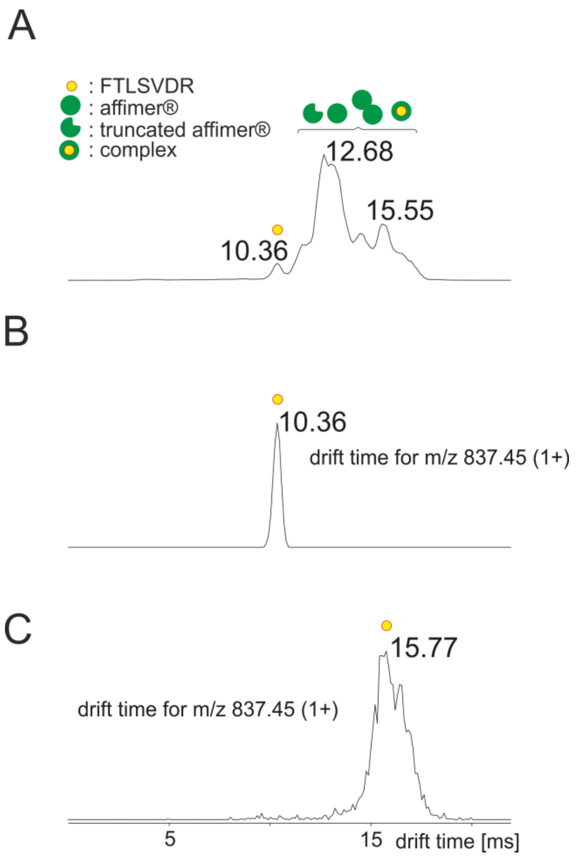

Figure S9: Ion mobility drift time plot of the FTLSVDR peptide incubated with the anti-Pertuzumab affimer 00557_709097.
The molar ratio of anti-Pertuzumab affimer 00557_709097 to the FTLSVDR peptide from the Pertuzumab heavy chain was 1 to 2. Drift times are given for ions from the anti-Pertuzumab affimer 00557_709097 (monomeric full-length affimer (green filled circle, ***I***), the dimeric full-length affimer (green filled circle tandem, ***II***), and the monomeric truncated affimer (green filled circle with rectangular cutout, ***III***), and for peptide FTLSVDR (amino acid range 68-74 from Pertuzumab heavy chain (HC); yellow filled circle). Complex ions are indicated with yellow filled circles centered within green filled circles. Traces were smoothed using the Savitzky Golay algorithm applying a smooth window of 2 channels and 2 smooth cycles. **A:** Drift time plot for all ions at Transfer collision cell voltage difference of 2V. **B:** Drift time plot for the ion with m/z value of 837.47 (singly protonated FTLSVDR peptide) at Transfer collision cell voltage difference of 2 V. **C:** Drift time plot for ion with m/z value 837.47 at Transfer collision cell voltage difference of 75 V.

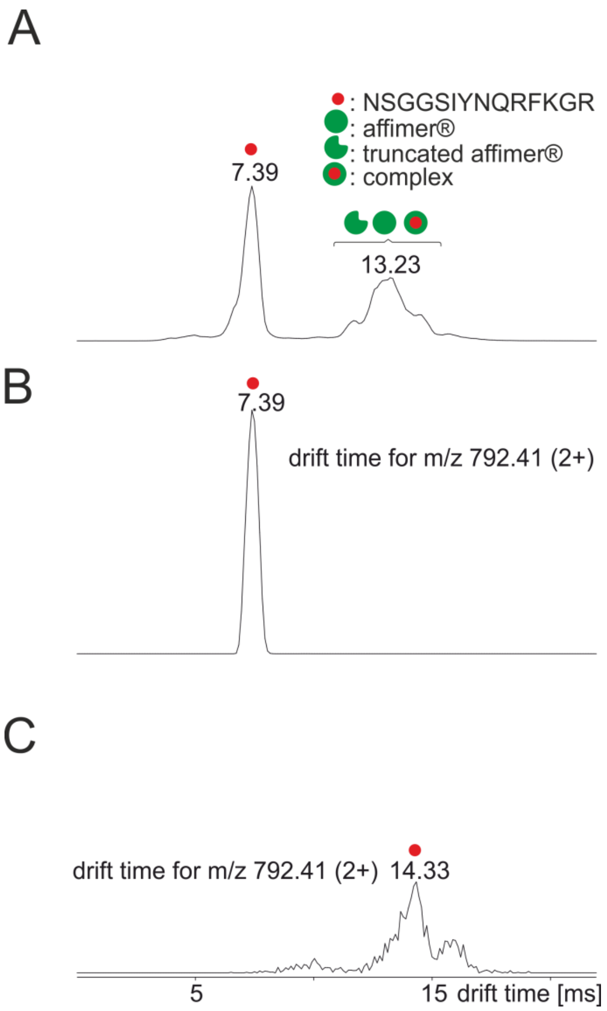

Figure S10: Ion mobility drift time plot of the NSGGSIYNQRFKGR peptide incubated with the anti-Pertuzumab affimer 00557_709097.
The molar ratio of anti-Pertuzumab affimer 00557_709097 to the NSGGSIYNQRFKGR peptide was 1 to 2. Drift times are given for ions from the anti-Pertuzumab affimer 00557_709097 (monomeric full-length affimer (green filled circle, ***I***), the dimeric full-length affimer (green filled circle tandem, ***II***), and the monomeric truncated affimer (green filled circle with rectangular cutout, ***III***), and for peptide NSGGSIYNQRFKGR (amino acid range 54-67 from Pertuzumab heavy chain (HC); red filled circle). Complex ions are indicated with red filled circles centered within green filled circles. Traces were smoothed using the Savitzky Golay algorithm applying a smooth window of 2 channels and 2 smooth cycles. **A:** Drift time plot for all ions at Transfer collision cell voltage difference of 2V. **B:** Drift time plot for ion with m/z value of 792.41 (doubly protonated peptide NSGGSIYNQRFKGR) at Transfer collision cell voltage difference of 2 V. **C:** Drift time plot for ion with m/z value 792.41 at Transfer collision cell voltage difference of 75 V.

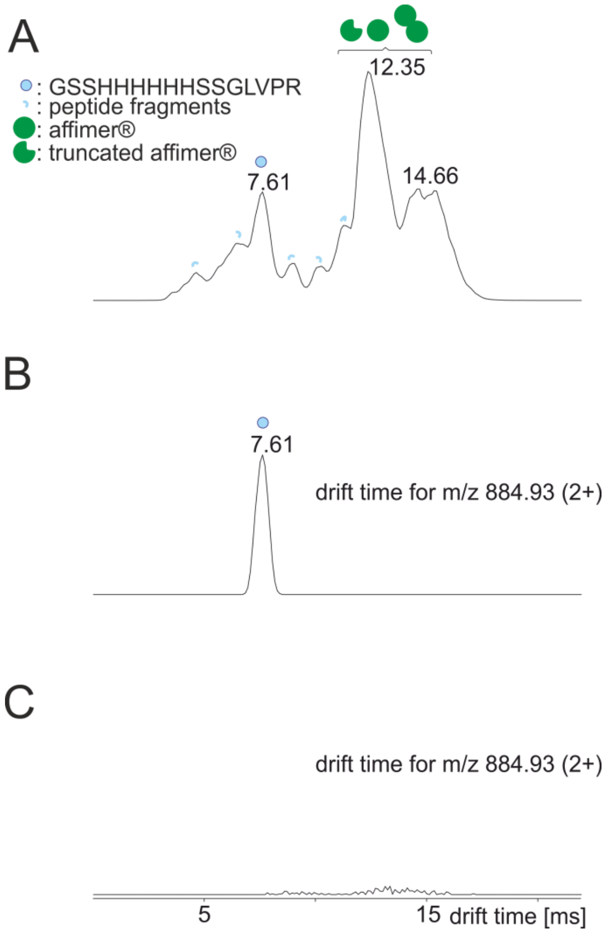

Figure S11: Ion mobility drift time plot of the GSSHHHHHHGLVPR His-tag peptide incubated with the anti-Pertuzumab affimer 00557_709097.
The molar ratio of anti-Pertuzumab affimer 00557_709097 to the GSSHHHHHHGLVPR peptide was 1 to 2. Drift times are given for ions from the anti-Pertuzumab affimer 00557_709097 (monomeric full-length affimer (green filled circle, ***I***), the dimeric full-length affimer (green filled circle tandem, ***II***), and the monomeric truncated affimer (green filled circle with rectangular cutout, ***III***), and for the His-tag peptide GSSHHHHHHGLVPR (light blue filled circle). Traces were smoothed using the Savitzky Golay algorithm applying a smooth window of 2 channels and 2 smooth cycles. **A:** Drift time plot for all ions at Transfer collision cell voltage difference of 2V. **B:** Drift time plot for ion with m/z value of 884.93 (doubly protonated His-tag peptide) at Transfer collision cell voltage difference of 2 V. **C:** Drift time plot for ion with m/z value 884.93 at Transfer collision cell voltage difference of 75 V.

Supplemental Tables

Table S1: Ion charge states, m/z values, intensities, and experimentally determined molecular masses of the complex consisting of Pertuzumab with two affimer monomers (VI) [a)].

z	m/z	intensity [b)]	MW (exp.)
31+	5661.47	1299	175474.57
30+	5848.81	3761	175434.30
29+	6051.06	4906	175451.74
28+	6265.64	3968	175409.92
27+	6499.06	2471	175447.62
26+	6748.52	1292	175435.52
	mean MW		175442.28 ± 21.5
28.6+	mean charge		

a) determined at ΔCV 60; see figure 1.
b) arbitrary units

Table S2: Ion charge states, m/z values, intensities, and experimentally determined molecular masses of the complex consisting of Pertuzumab with one affimer monomer (V) [a)].

z	m/z	intensity [b)]	MW (exp.)
29+	5579.95	315	161789.55
28+	5779.66	949	161802.48
27+	5996.87	1577	161888.49
26+	6224.83	1641	161819.58
25+	6472.56	1075	161789.00
	mean MW		161817.82 ± 41.42
26.6+	mean charge		

a) determined at ΔCV 60; see figure 1.
b) arbitrary units

Table S3: Ion charge states, m/z values, intensities, and experimentally determined molecular masses of Pertuzumab (IV) [a)].

z	m/z	intensity [b)]	MW (exp.)
27+	5486.95	1084	148120.65
26+	5697.99	2888	148121.74
25+	5924.91	4306	148097.75
24+	6172.40	3287	148113.60
23+	6440.27	1437	148103.21
	mean MW		148111.39 ± 10.62
24.9+	mean charge		

a) determined at ΔCV 60; see figure 1.
b) arbitrary units

Table S4: Ion charge states, m/z values, intensities, and experimentally determined molecular masses of the affimer monomer (I) [a)].

z	m/z	intensity [b)]	MW (exp.)
12+	1138.12	515	13645.44
11+	1241.44	1944	13644.84
10+	1365.54	4257	13645.40
9+	1517.22	11524	13645.98
8+	1706.67	4077	13645.36
7+	1950.56	689	13646.92
	mean MW		13645.66 ± 0.72
9.2+	mean charge		

a) determined at ΔCV 60; see figure 1.
b) arbitrary units

Table S5: Ion charge states, m/z values, intensities, and experimentally determined molecular masses of the affimer dimer (II) [a].

z	m/z	intensity [b]	MW (exp.)
21+	1301.16	314	27303.36
20+	1365.54	4257	27290.80
19+	1437.41	5229	27291.79
18+	1517.22	11524	27291.96
17+	1606.40	5798	27291.80
16+	1706.67	4077	27290.72
15+	1820.50	1301	27292.50
14+	1950.56	689	27293.84
13+	2100.49	215	27293.37
	mean MW		27293.35 ± 3.89
17.8+	mean charge		

a) determined at ΔCV 60; see figure 1.
b) arbitrary units

Table S6: Ion charge states, m/z values, intensities, and experimentally determined molecular masses of the truncated affimer monomer (III) [a].

z	m/z	intensity [b]	MW (exp.)
9+	1379.28	1907	12404.52
8+	1551.63	1799	12405.04
7+	1773.10	1021	12404.70
6+	2068.62	n.d.	12405.72
	mean MW		12405 ± 0.53
8.2+	mean charge		

a) determined at ΔCV 60; see figure 1.
b) arbitrary units

Table S7: Ion charge states, m/z values, intensities, and experimentally determined molecular masses of the truncated affimer dimer (VII) [a].

z	m/z	intensity [b]	MW (exp.)
13+	1810.49	601	23523.37
12+	1961.33	993	23523.96
11+	2139.34	615	23521.74
10+	2353.27	204	23522.70
	mean MW		23522.94 ± 0.95
12.0+	mean charge		

a) determined at ΔCV 60; see figure 1.
b) arbitrary units

Table S8: Protein ion intensities at measured collision cell voltage differences for measurement 1 of affimer 00557_709097 incubated with Pertuzumab.

ΔCV [V]	protein / complex [a]						
	truncated affimer (III)	affimer monomer (I)	Truncated affimer dimer	affimer dimer (II)	Pertuzumab (IV)	Pertuzumab + 1 affimer monomer (V)	Pertuzumab + 2 affimer monomers (VI)
2	n.d. [b]	n.d. [b]	n.d. [b]	n.d. [b]	22552	21081	30186
15	n.d. [b]	8139	n.d. [b]	n.d. [b]	229453	123367	332028
20	n.d. [b]	22587	n.d. [b]	n.d. [b]	167207	89045	321265
30	n.d. [b]	42133	n.d. [b]	n.d. [b]	348814	229900	564774
40	23168	90148	n.d. [b]	83558	320631	201844	602885
50	32056	245825	66346	380966	401136	209145	618865
60	58626	452034	114632	1295660	579104	189812	575729
70	107447	670342	191239	2230785	706622	220437	727978
80	182994	824306	226235	3590127	699719	164376	670724
90	229833	1036196	260373	4494586	696721	246100	965194

a) Roman numerals according to Figure 1. Unitless number.
b) n.d.: not determined, ion signal not present

Table S9: Protein ion intensities at measured collision cell voltage differences for measurement 2 of affimer 00557_709097 incubated with Pertuzumab.

ΔCV [V]	protein/complex a)						
	truncated affimer (III)	affimer monomer (I)	truncated affimer dimer	affimer dimer (II)	Pertuzumab (IV)	Pertuzumab + 1 affimer monomer (V)	Pertuzumab + 2 affimer monomers (VI)
2	n.d. b)	n.d. b)	n.d. b)	n.d. b)	48994	23576	80580
15	n.d. b)	14593	n.d. b)	26946	1092293	297775	1880182
20	n.d. b)	20021	n.d. b)	35657	552054	474910	1063679
30	n.d. b)	43543	n.d. b)	35248	722351	313323	2203527
40	36412	220384	29058	141521	1048765	444582	1297011
50	133824	485989	64991	921088	1563516	327095	2293527
60	176522	970922	143105	2662213	1331230	419534	1738065
70	65567	1494501	206241	4765817	2362325	496962	3263791
80	116800	1683159	260781	7400605	2459131	359917	1672313
90	92502	1868824	279150	9037523	2473329	456793	1661711

a) Roman numerals according to Figure 1. Unitless number. b) n.d.: not determined, ion signal not present

Table S10: Assigned Pertuzumab peptide ions upon tryptic digestion and desalting and unassigned peptide ion signals a).

m/z (exp.) / z	m/z (calc.) / z	amino acid range b)	partial sequence
941.50 / 2+	941.51 / 2+	HC 1-19	EVQLVESGGGLVQPGGSLR
952.48 / 2+	952.51 / 2+	HC 1-19 + Na	EVQLVESGGGLVQPGGSLR
1088.51 / 2+	1088.52 / 2+	HC 44-63	GLEWVADVNPNSGGSIYNQR
1099.51 / 2+	1099.52 / 2+	HC 44-63 + Na	GLEWVADVNPNSGGSIYNQR
**837.44 / 1+**	**837.45 / 1+**	**HC 68-74**	**FTLSVDR**
1186.65 / 1+	1186.68 / 1+ c)	HC 124-135	GPSVFPLAPSSK
661.34 / 2+	661.36 / 2+ c)	HC 136-149	STSGGTAALGCLVK
1321.68 / 1+	1321.71 / 1+ c)	HC 136-149	STSGGTAALGCLVK
1422.73 / 2+	1422.76 / 2+ c)	HC 225-250	THTCPPCPAPELLGGPSVFLFPPKPK
835.43 / 1+	835.43 / 1+	HC 251-257	DTLMISR
1070.00 / 2+	1070.02 / 2+	HC 258-276	TPEVTCVVVDVSHEDPEVK
1080.99 / 2+	1081.02 / 2+	HC 258-276 + Na	TPEVTCVVVDVSHEDPEVK
839.40 / 2+	839.40 / 2+	HC 277-290	FNWYVDGVEVHNAK
850.39 / 2+	850.40 / 2+	HC 277-290 + Na	FNWYVDGVEVHNAK
904.51 / 2+	904.51 / 2+	HC 304-319	VVSVLTVLHQDWLNGK
838.50 / 1+	838.52 / 1+ c)	HC 329-336	ALPAPIEK
643.84 / 2+	643.85 / 2+ c)	HC 347-357	EPQVYTLPPSR
1286.67 / 1+	1286.70 / 1+ c)	HC 347-357	EPQVYTLPPSR
1161.63 / 1+	1161.63 / 1+	HC 363-372	NQVSLTCLVK
1272.57 / 2+	1272.60 / 2+ c)	HC 373-394	GFYPSDIAVEWESNGQPENNYK
937.45 / 2+	937.46 / 2+	HC 395-411	TTPPVLDSDGSFFLYSK
948.44 /2+	948.46 /2+	HC 395-411 + Na	TTPPVLDSDGSFFLYSK
660.36 / 1+	660.38 / 1+ c)	HC 442-448	SLSLSPG
939.95 / 2+	939.97 / 2+ c)	LC 1-18	DIQMTQSPSSLSASVGDR
981.49 / 2+	981.51 / 2+	LC 25-42	ASQDVSIGVAWYQQKPGK
1051.56 / 2+	1051.57 / 2+	LC 108-126	RTVAAPSVFIFPPSDEQLK
973.51 / 2+	973.52 / 2+	LC 109-126	TVAAPSVFIFPPSDEQLK
899.45 / 2+	899.45 / 2+	LC127-142	SGTASVVCLLNNFYPR
560.32 / 1+	560.34 / 1+ c)	LC 146-149	VQWK
1068.47 / 2+	1068.49 / 2+	LC 150-169	VDNALQSGNSQESVTEQDSK
1079.47 / 2+	1079.49 / 2+	LC 150-169 + Na	VDNALQSGNSQESVTEQDSK
1502.76 / 1+	1502.79 / 1+ c)	LC 170-183	DSTYSLSSTLTLSK
938.47 / 2+	938.47 / 2+	LC 191-207	VYACEVTHQGLSSPVTK
949.47 / 2+	949.47 / 2+	LC 191-207 + Na	VYACEVTHQGLSSPVTK
842.49 / 1+	842.51 / 1+	Trypsin 108-115	VATVSLPR
869.50 / 1+	n.a. d)	n.a. d)	n.a. d)
886.52 / 1+	n.a. d)	n.a. d)	n.a. d)
913.51 / 1+	n.a. d)	n.a. d)	n.a. d)
930.54 / 1+	n.a. d)	n.a. d)	n.a. d)
960.46 / 2+	n.a. d)	n.a. d)	n.a. d)
969.46 / 3+	n.a. d)	n.a. d)	n.a. d)
1018.59 / 1+	n.a. d)	n.a. d)	n.a. d)
1032.57 / 1+	n.a. d)	n.a. d)	n.a. d)
1150.68 / 1+	n.a. d)	n.a. d)	n.a. d)

a) see figure 3A. b) HC: heavy chain, LC: light chain c) spectrum not shown d) n.a.: not assigned

Table S11: Molecular information on synthetic peptides.

peptide sequence	amino acid range [a)]	m/z (calc.) / z	m/z (exp.) / z
NSGGSIYNQRFKGR	HC 54-67	528.61 / 3+	528.59 / 3+
		792.41 / 2+	792.38 / 2+
FTLSVDR	HC 68-74	837.45 / 1+	837.42 / 1+
NSGGSIYNQRFKGR FTLSVDR	HC 54-74	801.42 / 3+	801.38 / 3+
		1201.62 / 2+	1201.56 / 2+
GSSHHHHHHSSGLVPR	n.a. [b)]	590.29 / 3+	590.28 / 3+
		884.93 / 2+	884.90 / 2+

a) HC: heavy chain. b) not applicable.
